# Association of rivaroxaban plasma trough concentrations with clinical characteristics and outcomes

**DOI:** 10.3389/fphar.2025.1563745

**Published:** 2025-03-18

**Authors:** Huizhen Wu, Qiaoling Yu, Panpan Jin, Lijing Huo, Jing An

**Affiliations:** ^1^ Department of Pharmacy, Hebei Key Laboratory of Clinical Pharmacy, Hebei General Hospital, Shijiazhuang, China; ^2^ Graduate School, Hebei Medical University, Shijiazhuang, China; ^3^ Department of Laboratory, Hebei General Hospital, Shijiazhuang, China

**Keywords:** rivaroxaban, therapeutic drug monitoring, impact factors, clinical safety events, real-word data

## Abstract

**Background:**

Rivaroxaban use has increased significantly among older adults; however, no definitive plasma concentration thresholds for bleeding or thrombosis have been established. However, dose adjustments for this population remain controversial.

**Methods:**

Between January 2022 and August 2023, we analyzed trough plasma samples from hospitalized patients treated with rivaroxaban for at least three consecutive days. Clinical data, including demographics, comorbidities, and adverse events, were extracted from electronic medical records. The plasma concentrations of rivaroxaban were measured using liquid chromatography-tandem mass spectrometry (LC-MS/MS). Statistical analyses were performed to identify factors influencing rivaroxaban exposure and clinical outcomes.

**Results:**

Among 360 plasma samples analyzed (55% male; median age: 72 years), age (P = 0.042) and renal function (P = 0.002) were significant predictors of rivaroxaban concentration-to-dose ratio. Bleeding events were associated with higher trough concentrations (median: 81.85 ng/mL in the bleeding group vs. 26.80 ng/mL in others; P < 0.001) and were more common in patients with malignancies or prior bleeding history. Thrombotic events occurred predominantly in older patients with a history of stroke (P < 0.05). Patients who died were older and had higher CHA2DS2-VASc scores (P < 0.05), prolonged prothrombin times (P < 0.001), and multiple comorbidities.

**Conclusion:**

Routine monitoring of rivaroxaban plasma concentrations may improve safety in older adults with multiple comorbidities or impaired hepatic, renal, or coagulation functions. Further research is required to establish specific therapeutic thresholds for bleeding and thrombosis.

## 1 Introduction

Rivaroxaban, an anti-Xa inhibitor, is effective in preventing stroke in patients with non-valvular atrial fibrillation (NVAF) and in treating pulmonary embolism or deep vein thrombosis (DVT) (Guimaraes et al., 2020; Fredenburgh and Weitz, 2023). In recent years, it has primarily replaced warfarin in many clinical applications, except for patients with mechanical valves (Fredenburgh and Weitz, 2023). Several clinical trials have demonstrated that rivaroxaban is non-inferior to warfarin in preventing stroke or systemic embolism (Carnicelli et al., 2022; Larsen et al., 2017; Patel et al., 2011), and can be administered at fixed doses without frequent monitoring (Fredenburgh and Weitz, 2023). However, real-world cohort studies have revealed considerable inter-individual variability in the plasma concentrations of rivaroxaban (Toorop et al., 2022). Although current evidence suggests a possible link between elevated rivaroxaban levels, genetic factors, race, and bleeding events (Xiang et al., 2023; Wong et al., 2014; Lin et al., 2023), defining specific thresholds for bleeding and thrombosis remains challenging.

To date, no definitive therapeutic window for rivaroxaban has been established, and considerable controversy surrounds the management of patients who experience bleeding, leading to potential safety concerns (Albaladejo et al., 2017). Older patients, who often have multiple comorbidities and polypharmacy, are at a higher risk of both bleeding and thrombosis than younger individuals (Tzoran et al., 2018). Research further indicates that older patients with acute venous thromboembolism (VTE) are at an increased risk of recurrent VTE and have higher case-fatality rates (Lauber et al., 2018). Rivaroxaban is frequently prescribed for anticoagulation in this population owing to fewer drug-drug interactions (DDIs) and adverse events than those commonly associated with warfarin (Larsen et al., 2017). However, rivaroxaban is not exempt from drug-drug or drug-food interactions. Recent studies have identified potential interactions with common clinical medications, foods, and herbs, such as levetiracetam and valproic acid (Paciullo et al., 2020; Goldstein et al., 2023; Grześk et al., 2021; Vazquez, 2018).

Therefore, ensuring the safe use of rivaroxaban is crucial, particularly in older patients. This study aimed to identify factors associated with rivaroxaban plasma concentrations and clinical safety events, and to analyze common concerns in real-world clinical practice.

## 2 Methods

### 2.1 Study design and patients

This single-center cohort study was conducted at Hebei General Hospital and enrolled patients who received rivaroxaban between January 2022 and August 2023. The inclusion criteria were as follows: (1) the patient was an inpatient, (2) the patient had received rivaroxaban for at least three consecutive days to achieve steady-state plasma concentrations, and (3) the steady-state trough concentration of rivaroxaban was monitored at least once. The exclusion criteria were as follows: (1) a measured rivaroxaban plasma concentration below 1 ng/mL or above 500 ng/mL and (2) incomplete or missing clinical test data.

Patient demographics and laboratory test results were collected from the electronic medical records. The following information was collected: age, sex, body mass index (BMI), rivaroxaban dose, comorbidities, anticoagulation indication, medical history (e.g., hypertension, diabetes mellitus, stroke, and bleeding history), liver and renal function parameters (e.g., estimated glomerular filtration rate [eGFR]), and coagulation parameters (e.g., prothrombin time [PT], activated partial thromboplastin time [APTT], thrombin time [TT], and D-dimer). Renal function was monitored using eGFR, and hepatic function was assessed according to the Child-Pugh grade (Lin et al., 2021; Kubitza et al., 2013). Kidney function was staged according to the kidney Disease: Improving Global Outcomes (KDIGO) guidelines, whereas BMI was classified using the World Health Organization (WHO) standard (Kidney Disease: Improving Global Outcomes (KDIGO) CKD Work Group, 2024).

Coagulation parameters included PT, APTT, TT, and D-dimer levels. CHA2DS2-VASc (Vera Sainz et al., 2022) and HAS-BLED (Liu et al., 2023) scores were recorded to assess each patient’s risk of stroke and bleeding, respectively. The clinical outcomes included bleeding, acute stroke, and all-cause mortality during rivaroxaban use. Major bleeding events were defined as life-threatening hemorrhages (e.g., cerebral hemorrhage, hemorrhagic shock, and severe gastrointestinal bleeding), a reduction in hemoglobin level >5 g/dL, or a requirement for ≥4 units of packed red blood cells (Schulman and Kearon, 2005). Other bleeding events included visible hemorrhage such as ecchymosis, epistaxis, or ophthalmorrhagia. Clinical outcomes were documented through telephone follow-up and review of readmission records.

This study was approved by the Ethics Committee of Hebei General Hospital (approval no. 2023329). As this investigation relied on medical records and biological specimens obtained from prior clinical diagnoses and treatments, a waiver of informed consent was granted following the Code for Quality Management of Pharmaceutical Clinical Trials of the People’s Republic of China, ICH-GCP, and other pertinent legal regulations.

### 2.2 Blood sampling and analysis

Since 2021, the hospital has offered a routine therapeutic drug monitoring (TDM) service for measuring rivaroxaban plasma concentrations. Serum samples were obtained from the hospital clinical pharmacy department as part of the TDM service. Based on prior pharmacokinetic data for rivaroxaban, trough concentrations were determined by collecting blood samples before the morning dose (approximately 24 h after administration) (Hindley et al., 2023).

Blood samples were drawn into blue-top tubes containing 3.8% sodium citrate as an anticoagulant and centrifuged at 3,500 r/min for 10 min on the collection day. The resulting serum was separated, stored at −20°C, and analyzed within 3 months. The rivaroxaban dose, dosing time, and blood sampling time were recorded for all the participants.

Protein precipitation was used to prepare plasma samples. Standard reference samples of rivaroxaban (Lot: 01001, purity: 98.7%), internal standard rivaroxaban-d4 (Lot: 02001-50, purity: 98.9%), and Quality Control (QC) solutions (Lot: H/M/L230825, purity: 98.7%) were purchased from Nanjing Pinsheng Pharmaceutical Company (China). The materials were brought to room temperature on the day of the assay. Specifically, 200 μL of the internal standard solution was added to both the working standards and QC dilutions. Eight standard solution concentrations (1.02, 2.56, 6.4, 16, 40, 100, 250, and 500 ng/mL) and three QC concentrations (10.7, 108, and 388 ng/mL) were prepared. After thawing at room temperature, 50 μL of plasma was mixed with 200 μL of the internal standard solution in an EP tube, vortexed for 5 min, and centrifuged at 12,000 rpm for 10 min at room temperature. Subsequently, 100 μL of the supernatant was collected and injected into the HPLC-MS/MS system.

Plasma concentrations were measured using a validated high-performance liquid chromatography-tandem mass spectrometry (HPLC-MS/MS) system consisting of an LC-20AD HPLC system coupled with an AB Sciex Quadrupole 3200MD triple quadrupole mass spectrometer equipped with an electrospray ionization (ESI) source in positive mode. Data acquisition and processing were performed using AB SCIEX Analyst 1.6.3 software (Applied Biosystems, Foster City, CA). Chromatographic separation was performed using an XBridge BEH C18 column (100 mm × 2.1 mm, 2.5 μm) at a flow rate of 0.4 mL/min and a column temperature of 40°C. The mobile phase was comprised of water (solvent A) and 0.1% formic acid in acetonitrile (solvent B). The total elution time was 6 min, with the following gradient profile: 0–2.4 min, 10%–98% B; 2.4–3.4 min, 98% B; and 3.4–6.0 min, 10% B.

Quantification was performed in the multiple-reaction monitoring (MRM) mode. The precursor-product ion transitions monitored were m/z 436.3 → 145.3 for rivaroxaban and m/z 440.3 → 145.0 for rivaroxaban-d4. The declustering potential was set to 100 V and the collision energy was set to 40 V. Nebulizer gas, dry gas, and curtain gas were applied at 55, 50, and 20 psi, respectively.

The linear range was 1–500 ng/mL (*R* ^2^ = 0.998) and the detection limit was 0.5 ng/mL. Intra- and inter-day relative standard deviations (RSDs) were within 15% for low, medium, and high QC samples, respectively. Rivaroxaban in biological samples remained stable for at least 24 h at room temperature, through three freeze-thaw cycles at −20°C, and during long-term storage at −20°C for up to 30 days. No significant interfering peaks were observed in this assay.

### 2.3 Statistical analysis

For normally distributed continuous variables, data are expressed as mean ± standard deviation (X¯ ± SD). Independent sample t-tests were used to compare group differences, and Pearson’s correlation was used for the correlation analysis. For non-normally distributed continuous variables, data are presented as medians with interquartile ranges [M, Q1-Q3]. Group comparisons were conducted using the Mann-Whitney U or Kruskal–Wallis H tests, while Spearman’s correlation was used for correlation analysis.

Categorical variables are reported as frequencies (n) and percentages (%). Categorical variables were compared between the groups using either Pearson’s chi-square test or Fisher’s exact test, as appropriate. Statistical significance was set at P < 0.05.

The Concentration-to-Dose Ratio (CDR) was calculated as the ratio of the trough plasma concentration to the daily dose (mg/day) of rivaroxaban. Univariate analysis was conducted to identify potential factors associated with CDR, including age, sex, BMI, kidney function, Child-Pugh grade, and concomitant medications. Variables with a p-value <0.05 in the univariate analysis were included in the multivariate linear regression model to adjust for potential confounders. As the data were continuous variables, we chose multiple linear regression for the multi-factor analysis. P < 0.05 was considered statistically significant.

All statistical analyses were performed using IBM SPSS Statistics version 27.0 (IBM Corporation, Armonk, NY, United States of America) and Origin 2021 (OriginLab Corporation, Northampton, MA, United States of America).

## 3 Results

### 3.1 Patient characteristics

A total of 367 plasma samples were collected. One sample exceeded the upper limit of quantification and six were below the lower limit. Consequently, 360 samples from 274 patients were included in this analysis. The demographic and clinical characteristics of the study population are summarized in Table 1.

**TABLE 1:** Patient characteristics.

Characteristics	360 samples (from 274 patients)
Gender, male/female (n, %)	198/162 (55%/45%)
Age (year), median (IQR)	72 (63, 85)
≤18, n (%)	3 (0.8%)
19-64, n (%)	96 (26.52%)
≥65, n (%)	261 (72.5%)
BMI (kg/m^2^), median (IQR)	24.89 (22.87, 27.73)
<18.5, n (%)	11 (3.05%)
18.5–23.9, n (%)	97 (26.94%)
24-27.9, n (%)	113 (31.39%)
≥28, n (%)	71 (19.72%)
Rivaroxaban plasma concentration (ng/mL), median (IQR)	30.4 (11.03, 64.85)
Rivaroxaban dose (mg/d); median (IQR)	15 (10, 20)
Once-daily, n (%)	324 (90.06%)
Twice-daily, n (%)	36 (9.94%)
Anticoagulation indication, n (%)	
Atrial fibrillation	109 (30.28%)
Venous thromboembolism	148 (41.11%)
Atrial fibrillation and venous thromboembolism	61 (16.94%)
Other	42 (11.67%)
Kidney function (mL/min), median (IQR)	82.59 (60.85, 95.37)
90–120 mL/min, n (%)	122 (33.7%)
60–89 mL/min, n (%)	138 (38.33%)
30–59 mL/min, n (%)	67 (18.5%)
15–29 mL/min, n (%)	14 (3.87%)
<15 mL/min, n (%)	2 (0.55%)
Child-Pugh grade, n (%)	
A	272 (75.56%)
B	30 (8.33%)
C	2 (0.55%)
Concomitant P-gp/CYP3A4 inducer use, n (%)	2 (0.55%)
Sodium phenobarbital, n (%)	1 (0.36%)
Levetiracetam, n (%)	1 (0.36%)
Concomitant P-gp/CYP3A4 inhibitor use, n (%)	64 (17.96%)
Tolvaptan, n (%)	23 (8.39)
Amiodarone, n (%)	17 (6.2)
Voriconazole and Fluconazole, n (%)	12 (4.38)
Ritonavir, n (%)	7 (2.55)
Almonertinib, Anlotinib and Osimertinib, n (%)	5 (1.83)
Linagliptin, n (%)	5 (1.83)
Tacrolimus, n (%)	3 (1.09)
Ciclosporin, n (%)	1 (0.36)
Rifaximin, n (%)	1 (0.36)
Concomitant herbal medicines, n (%)	274 (76.11%)
Concomitant antiplatelet and heparin-like medicines, n (%)	157 (43.61%)
Concomitant proton pump inhibitors, n (%)	150 (41.67%)
Note	Voriconazole and ritonavir were co-administered in one patient, linagliptin and tolvaptan in two, tolvaptan and amiodarone in three, and voriconazole and tacrolimus in three

The median age of the patients was 72 years, with 72.5% of the patients aged 65 years or older. Male patients accounted for 55% of the sample. The median BMI was 24.89 kg/m^2^, with 31.39% of patients classified as overweight (24–27.9 kg/m^2^) and 19.72% classified as obese (≥28 kg/m^2^). The median eGFR for renal function was 82.59 (60.85, 95.37) mL/min. Mild renal impairment (eGFR 60–89 mL/min) was observed in 38.33% of the patients, while 22.92% had moderate or severe renal impairment (eGFR <60 mL/min), including two patients with end-stage renal disease (eGFR <15 mL/min).

Most patients had normal liver function, with 75.56% classified as Child-Pugh grade A. However, 30 patients (8.33%) were classified as grade B, and 2 patients (0.55%) were classified as grade C.

The median plasma concentration of rivaroxaban was 30.4 (11.03, 64.85) ng/mL, and the median daily dose was 15 mg (10, 20 mg). Most patients (90.06%) received a once-daily regimen, whereas the remaining 9.94% received a twice-daily regimen.

Rivaroxaban was prescribed for various indications: atrial fibrillation (30.28%), venous thromboembolism (41.11%), both conditions concurrently (16.94%), and other indications (11.67%).

In terms of concomitant medications, two patients (0.55%) received cytochrome P450 (CYP)/P-glycoprotein (P-gp) inducers, while 64 patients (17.96%) received CYP/P-gp inhibitors. Additionally, 157 patients (43.61%) were administered antiplatelet or heparin-like drugs, 150 (41.67%) received proton pump inhibitors (PPIs), and 274 (76.11%) used herbal remedies concurrently with rivaroxaban therapy.

### 3.2 Factors associated with rivaroxaban plasma trough concentrations

#### 3.2.1 Correlation of rivaroxaban plasma trough concentrations with dose and frequency

**FIGURE 1:**
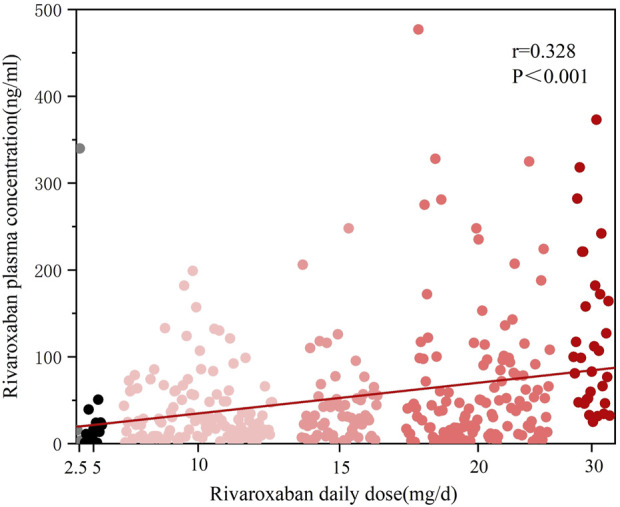
Scatter plot of sample rivaroxaban plasma concentration distribution by dose group.

This study observed a significant positive correlation between rivaroxaban plasma trough concentrations and the administered daily dose in all patients (r = 0.328, P < 0.001, Figure 1).

**FIGURE 2:**
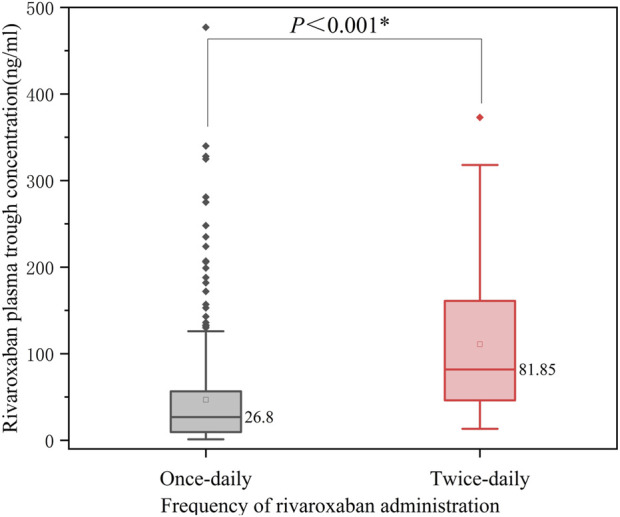
Effect of dosing frequency on the trough concentration of rivaroxaban.

When comparing dosing regimens, we found that patients receiving twice-daily rivaroxaban (n = 36) had significantly higher plasma trough concentrations than those receiving a once-daily regimen (n = 324) (81.85 [46.15, 162.50] ng/mL vs. 26.80 [9.51, 56.62] ng/mL, P < 0.001, Figure 2). Furthermore, the correlation between the daily dose and plasma concentration was more pronounced in the twice-daily group (r = 0.388, P = 0.02) than in the once-daily group (r = 0.206, P < 0.001). These findings highlight the potential influence of dosing frequency on plasma levels of rivaroxaban. The detailed results are presented in Table 2. Despite these findings, the relatively small sample size in the twice-daily dosing group limited the robustness of these comparisons.

**TABLE 2:** Correlation between rivaroxaban trough concentrations, dose, and dosing frequency.

Group	Number (n)	Trough levels (ng/mL), median (IQR)	P (trough levels)	Daily dose (mg/d), median (IQR)	r (Correlation)	P (correlation)
Once Daily	324	26.80 (9.51, 56.62)	<0.001	15 (10, 20)	0.206	<0.001
Twice daily	36	81.85 (46.15, 162.50)		15 (15, 15)	0.388	0.02
Total	360	30.40 (11.03, 64.85)		15 (10, 20)	0.328	<0.001

#### 3.2.2 Correlation of rivaroxaban trough plasma concentrations with patient characteristics and concomitant medications

**FIGURE 3:**
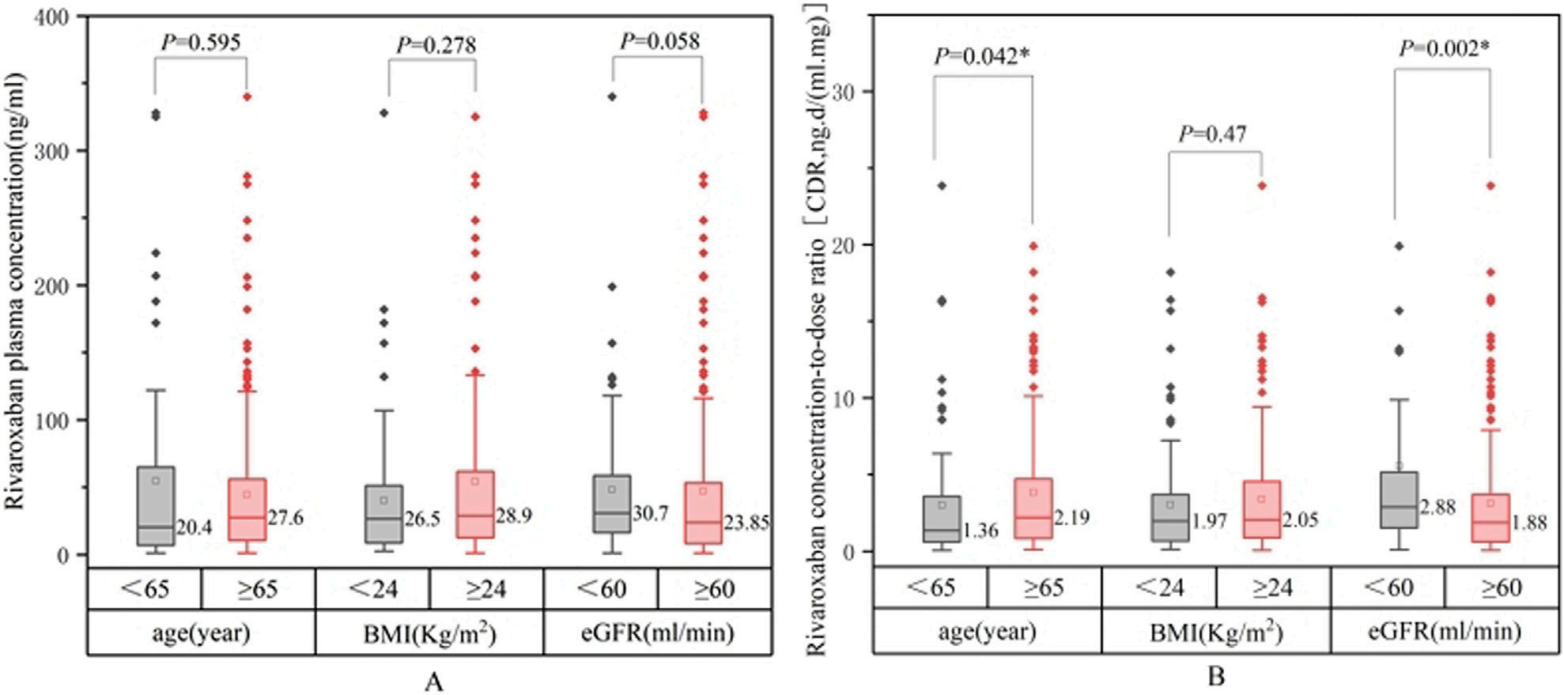
Comparison between rivaroxaban plasma trough levels **(A)** or concentration-dose ratio CDR **(B)**.

**FIGURE 4:**
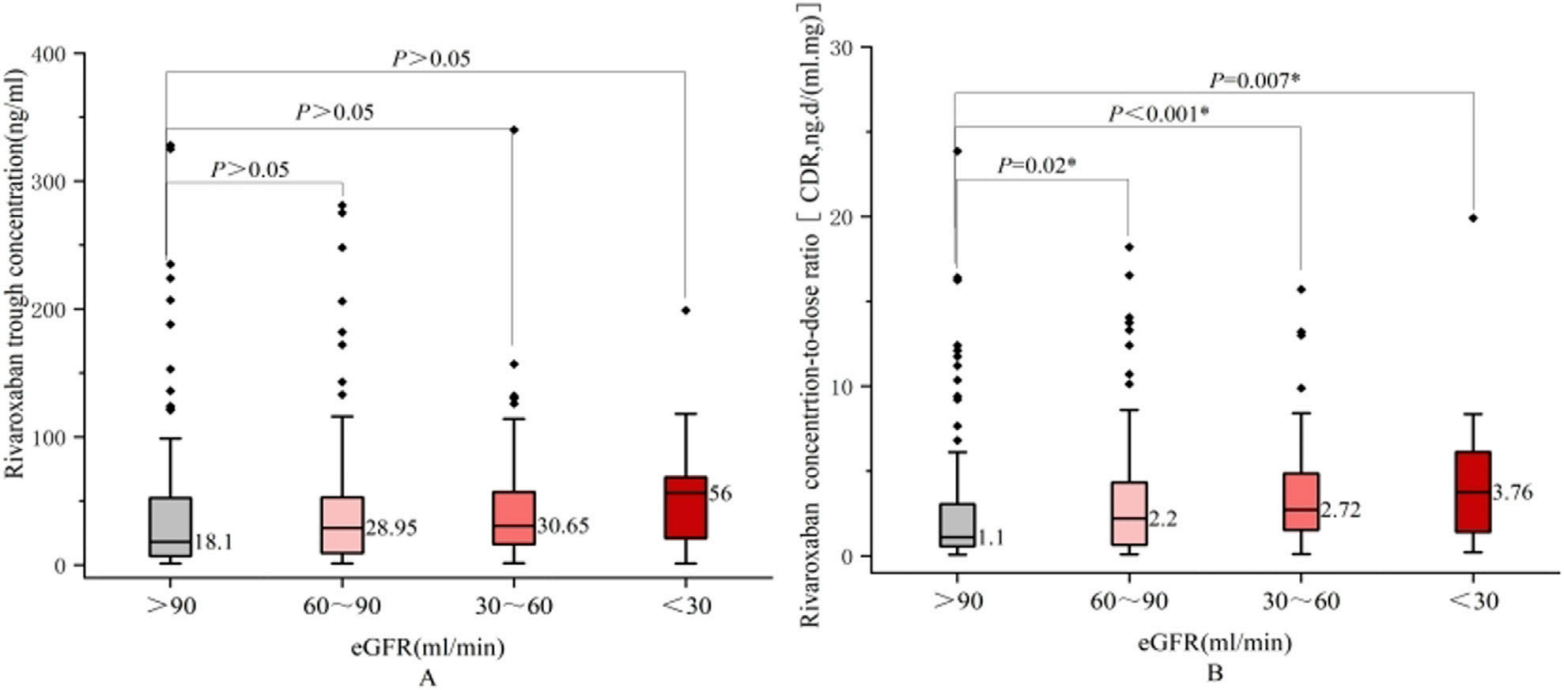
Comparison between rivaroxaban plasma trough levels **(A)** or CDR **(B)** and eGFR Grade.

Comparisons of the rivaroxaban trough plasma concentrations across patients with varying baseline characteristics are summarized in Table 3. Figures 3, 4 show the relationship between rivaroxaban plasma trough levels or CDR and variables, such as age, BMI, and eGFR. In univariate analysis, age and renal function were significantly associated with rivaroxaban trough plasma levels (P < 0.05), whereas sex and hepatic function were not. Female patients showed a trend toward higher rivaroxaban exposure than male patients; however, this difference was not statistically significant. Similarly, patients with Child-Pugh grade B exhibited lower rivaroxaban levels than those with normal liver function. However, the difference was not significant, likely because of the small sample size of grade B patients (n = 25).

**TABLE 3:** Results of univariate analysis of rivaroxaban trough levels and CDR.

Characteristics	Group	Number (n)	Daily dose (mg/d) M (IQR)	Trough levels (ng/m) median (IQR) / ‾X± SD	CDR, ng.d/(mL.mg) Median (IQR)
Sex	Male	176	15(10,20)	21.40(8.03,52.20)	1.78(0.64,3.77)
	Female	148	15(10,20)	32.0(12.78,60.28)	2.46(0.81,4.57)
Age (year)	19–64	71	20(15,20)	20.40(6.82,65.00)	1.36(0.60,3.58)
	≥65	251	10(10,20)*	27.60(10.70,56.00)	2.19(0.83,4.73)*
BMI (kg/m^2^)	<18.5^1^	11	15(15,20)	67.33 ± 50.82	3.58(1.97,4.81)
	18.5–23.9^2^	88	10(10,20)	22.80(8.32,50.50)	1.70(0.62,3.68)
	24–27.9^3^	105	15(10,20)^(2,3)^*	33.80(12.8,67.85)	2.28(0.92,4.69)
	≥28^4^	55	20(10,20)^(2,4)^*	26.90(8.45,50.00)	1.88(0.57,3.58)
	<18.5	11	20(15,20)	31.0(22.30,96.20)	2.76(1.49,4.81)
	>18.5	248	15(10,20)	26.80(10.48,56.40)	2.0(0.74,4.03)
	<24	102	15(10,20)	26.50(8.64,52.18)	1.97(0.66,3.75)
	>24	157	15(10,20)*	28.90(11.90,63.80)	2.05(0.86,4.56)
Kidney function (eGFR,mL/min)	90–120^1^	101	20(10,20)^(1,2)^*	18.10(6.90,55.55)	1.1(0.55,3.06)
	60–89^2^	132	15(10,20)^(2,3)^*	28.95(9.11,53.05)	2.20(0.65,4.34)^(1,2)^*
	30–59^3^	62	10(10,15)^(1,3)^*	30.65(16.15,57.75)	2.72(1.53,4.93)^(1,3)^*
	<30^4^	15	10(10,15)^(1,4)^*	56.0(20.90,68.50)	3.76(1.39,6.12)^(1,4)^*
	<60	76	10(10,15)	30.70(16.30,59.45)	2.88(1.52,5.16)
	>60	234	15(10,20))**	23.85(8.24,53.82)	1.88(0.59,3.75)*
Child-Pugh grade	A	251	15(10,20)	26.90(10.80,58.80)	2.12(0.72,4.33)
	B	25	15(15,20)	21.40(8.97,50.95)	1.69(0.55,3.15)
P-gp/CYP3A4 inhibitor	non-Concomitant	258	15(10,20)	25.85(8.82,52.55)	2.0(0.67,4.1)
	Concomitant	64	15(10,20)	30.0(12.22,65.60)	2.56(1.0,4.91)
Herbal medicines	non-Concomitant	77	15(10,20)	26.90(12.20,64.35)	1.97(0.81,4.43)
	Concomitant	247	15(10,20)	26.50(9.40,56.40)	2.12(0.68,4.22)

Note: “M (IQR)” means the median (quartile); “(X ± SD” means the mean ± standard deviation.“*”, “P < 0.05” and “* *” means “P < 0.001”, a statistically significant difference.

CDR: concentration-dose ratio, IQR: interquartile range, BMI: body mass index, eGFR: estimated glomerular filtration.

Patients aged ≥65 years had significantly higher rivaroxaban trough levels and elevated CDR values (P = 0.042). Renal function was also a critical determinant, as patients with an eGFR <60 mL/min demonstrated significantly higher CDR values than those with an eGFR ≥60 mL/min (P = 0.002). CDR values increased with worsening renal impairment (P < 0.05), indicating a strong correlation between reduced eGFR and higher rivaroxaban exposure.

Although BMI was not significantly correlated with rivaroxaban levels or CDR in the univariate analysis (P = 0.135), subgroup analyses revealed nuanced trends. Patients with a BMI <18.5 kg/m^2^ tended to exhibit higher rivaroxaban plasma concentrations and CDR values than those with a BMI ≥18.5 kg/m^2^. In contrast, patients with BMI <24 kg/m^2^ had lower plasma concentrations than those with BMI ≥24 kg/m^2^. These trends suggest that extreme BMI values may influence rivaroxaban exposure, but statistical significance was not achieved, likely due to the small sample sizes in these subgroups; therefore, in subsequent multi-factor analyses, BMI was continuously included for analysis.

Patients receiving concomitant CYP3A4/P-gp inhibitors showed higher rivaroxaban trough levels than those without inhibitors or inducers; however, the differences were not statistically significant. Similarly, concurrent use of herbal medications did not significantly affect the plasma concentrations of rivaroxaban. Given the limited sample size, further analysis was not conducted for patients under 18 years of age (n = 2), those with Child-Pugh grade C (n = 1), and those receiving CYP3A4/P-gp inducers (n = 2).

The multifactorial analysis in Table 4 revealed that dosing frequency and BMI significantly affected rivaroxaban trough concentrations, with dosing frequency showing the strongest effect (β = 0.253, P < 0.001). BMI was also significantly associated with trough levels (β = 0.133, P = 0.022). In contrast, neither age nor eGFR was significantly correlated with trough concentration in this model.

**TABLE 4:** Results of multifactorial analysis of rivaroxaban trough levels and CDR.

Rivaroxaban	Factor	B	β	t	P	F	*R* ^2^ (adjusted)
Trough Levels	Frequency	56.779	0.253	4.192	<0.001	7.149	0.082
	Age	−6.625	−0.042	−0.673	0.502		
	eGFR	4.768	0.028	0.467	0.641		
	BMI	19.163	0.133	2.302	0.022		
CDR	Frequency	4.982	0.354	5.96	<0.001	9.448	0.109
	Age	0.55	0.059	0.963	0.336		
	eGFR	−0.033	−0.003	−0.053	0.958		
	BMI	0.495	0.055	0.964	0.336		

Note: B = unstandardized regression coefficient; β = standardized regression coefficient; t = t-value; P = significance level; F = F-statistic; *R* ^2^ (Adjusted) = adjusted coefficient of determination; eGFR, estimated glomerular filtration rate; BMI, body mass index; CDR, concentration-to-dose ratio.

Dosing frequency remained the primary determinant of CDR (β = 0.354, P < 0.001). Other variables, including BMI, age, and eGFR, were not significantly associated with the CDR in the multifactorial model. The differences between the univariate and multivariate results highlight the potential influence of confounding variables, which are better accounted for in multifactorial analyses, leading to a more accurate assessment of the independent effects of each factor.

### 3.3 Descriptive and statistical analyses of clinical outcomes

A total of 33 patients experienced hemorrhagic events during rivaroxaban therapy. Additionally, 12 patients developed cerebral infarction or a new systemic embolism, and 17 died. The median follow-up period was 28 days. Skin ecchymosis (n = 12) and gastrointestinal bleeding (n = 7) were the most common manifestations. Other hemorrhagic events included reduced hemoglobin, epistaxis, hematuria, and hematoma (n = 4 each); ophthalmorrhagia and hemoptysis (n = 3 each); hematochezia and oral hemorrhage (n = 2 each); and gingival bleeding, hematemesis, and intracranial hemorrhage (n = 1 each).

Among the bleeding cases, some patients experienced multiple hemorrhagic events. One patient exhibited sequential skin ecchymosis, hemoptysis, epistaxis, and ophthalmorrhagia. Another patient died after experiencing gastrointestinal bleeding, reduced hemoglobin levels, and hematoma, whereas a third patient died following intracranial hemorrhage, gastrointestinal bleeding, and hemoglobin decline. Of the 17 total deaths, 10 occurred in patients with a history of hemorrhage. Clinical management of bleeding events included discontinuation of rivaroxaban, administration of hemostatic agents (e.g., etamsylate and prothrombin), PPIs (e.g., esomeprazole), fresh frozen plasma, and red blood cell transfusions. A summary of bleeding events and their management is presented in Table 5.

**TABLE 5:** Clinical events and management.

Events	Samples (n)	Management
All bleeding	33	-
Skin ecchymosis	12	Administration of etamsylate, thrombin agent, and herbal preparations for blood replenishment; discontinuation of rivaroxaban
Haemoglobin reduction	4	Transfusion of blood or red blood cells
Gastrointestinal	7	Transfusion of fresh frozen plasma, platelets, or albumin; intravenous esomeprazole; administration of rebamipide or teprenone and thrombin agent; discontinuation of rivaroxaban
Gingival	1	Reduced rivaroxaban
Hematochezia	2	Administration of carbazochrome and pit viper hemagglutinin; suspended anticoagulation
Epistaxis	4	Discontinuation of rivaroxaban; nasal sponge stuffing
Ophthalmorrhagia	3	Administration of etamsylate
Hematuria	4	Administration of platelet-raising capsules; discontinuation of rivaroxaban
Intracranial	1	-
Hemoptysis	3	-
Haematemesis	1	-
Haematoma	4	-
Oral hemorrhage	2	Administration of prothrombin with gargle; administration of esomeprazole and herbal preparations for blood replenishment and hemostasis
New-onset venous thrombosis	12	Discontinuation of rivaroxaban; pump argatroban for anticoagulation; increased rivaroxaban dosage
All-cause death	17	-

Note: The symbol “-” indicates that a particular therapeutic measure was not indicated because it was either not documented in the medical record or no longer necessary.

**FIGURE 5:**
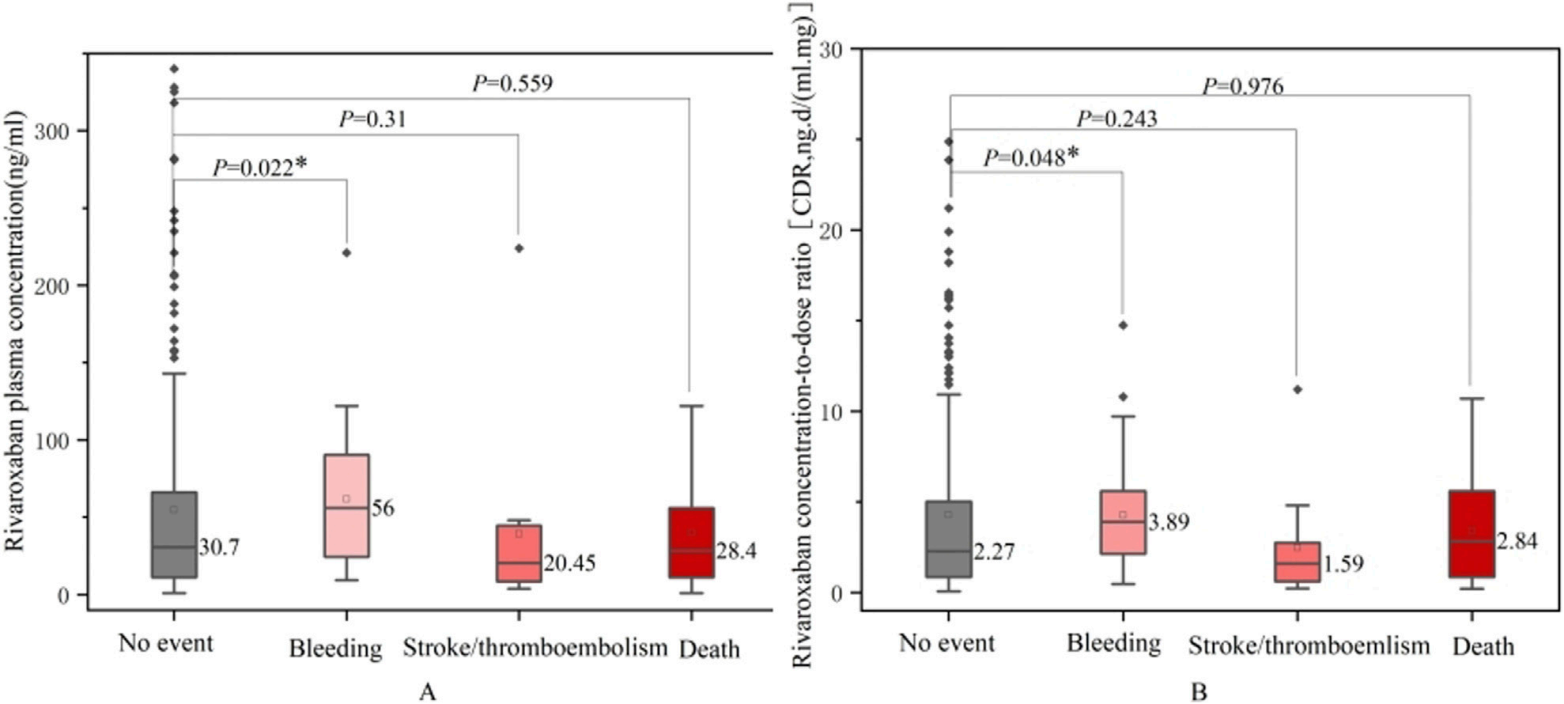
Comparison between rivaroxaban plasma trough levels **(A)** or CDR **(B)** and no event bleeding, stroke/thromboembolism and death groups.

To identify potential risk factors for adverse clinical outcomes, we compared rivaroxaban exposure, patient characteristics, and co-medications between those who experienced safety events (bleeding, thrombosis, or death) and those who did not (Table 6). Figure 5 compares rivaroxaban plasma trough levels and CDR across patient groups that experienced bleeding, thrombotic events, or death. Patients in the bleeding group exhibited significantly higher plasma concentrations of rivaroxaban and CDR values than those in the control group (P < 0.05). They also had a higher prevalence of history of bleeding and malignancy. Conversely, patients in the thrombotic group were older and had a greater prevalence of prior stroke than those in the normal group (P < 0.05). However, their rivaroxaban exposure was lower and the difference was insignificant.

**TABLE 6:** Comparison of basic characteristics between patients with and without clinical events.

Characteristics	Group	Normal	Bleeding	Stroke/Thromboembolism	All-cause death
	Number (n)	295	33	12	17
Indication	Non-valvular atrial fibrillation		14	6	9
	venous thrombus		22	5	10
	other		3	2	1
Rivaroxaban	Trough levels (ng/mL), M(IQR)	30.70 (11.10,66.20)	56.00 (22.60,90.80)*	20.45 (8.12,45.72)	28.40 (9.04,58.80)
	Daily dose(mg/d),M(IQR)	15(10,20)	15(10,20)	15(10,20)	10(10,15)*
	CDR,ng.d/(mL.mg), M(IQR)	2.27 (0.85,5.02)	3.89 (2.07,5.72)*	1.59 (0.53,2.79)	2.84 (0.70,5.85)
	Twice-daily, n(%)	31(10.51%)	3(9.09%)	0	1(5.88%)
	Low dose, n(%)	117(39.66%)	9(27.27%)	3(25.0%)	9(52.94%)
Patient characteristics	Age M(IQR)/±SD	72.00 (63.00,85.00)	70.00 (60.50,90.00)	81.00 ± 12.70*	91.00 (83.00,93.50)**
	≥65, n(%)	214(72.54%)	22(66.67%)	10(83.33%)	14(82.35%)
	Female, n(%)	135(45.76%)	14(42.42%)	4(33.33%)	6(35.29%)
	BMI, X±SD	25.57 ± 3.99	25.14 ± 3.80	25.37 ± 2.98	23.75 ± 3.91
	eGFR, M(IQR)/ ‾X±SD	85.18 (61.78,96.70)	81.53 ± 19.80	78.13 ± 18.60	74.64 ± 25.94
	PT, M(IQR)	12.20 (11.30,13.50)	12.80 (11.73,14.07)	11.90 (11.35,12.45)	13.50 (12.55,15.05)*
	Child-Pugh B, N (%)	17(5.76%)	3(9.09%)	1(8.33%)	4(23.53%)
Score	CHA2DS2VASc	4.00(2.00,5.00)	4.00(2.00,5.50)	4.33 ± 1.61	5.00(3.50,6.00)
	HAS-BLED	2.00(1.00,3.00)	2.00(1.50,3.00)	2.83 ± 1.03	3.00(2.00,3.50)
Concomitant disease	Bleeding/stroke history, n(%)	16 (5.42%)/78(26.44%)	14(42.42%)**	8(66.67)*	0/7(41.18%)
	Hypertension	150(50.85%)	18(54.54%)	8(66.67%)	15(88.24%)*
	Diabetes	70(23.73%)	12(36.36%)	3(0.25%)	4(23.53%)
	Tumour	23(7.80%)	10(30.30%)*	4(33.33%)	5(29.41%)
	Coronary heart	84(28.47%)	13(39.39%)	4(33.33%)	10(58.82%)*
	Heart failure	55(18.64%)	6(18.18%)	2(16.67%)	4(23.53%)
Concomitant	CYP3A4/P-gp inhibitor	53(17.97%)	7(21.21%)	4(33.33%)	3(17.65%)
	Herb medicine	225(76.27%)	25(75.76%)	10(83.33%)	13(76.47%)
	Proton pump inhibitor	122(41.36%)	18(54.54%)	3(25.00%)	4(23.53%)
	antiplatelet drug	126(42.71%)	16(48.48%)	5(41.67%)	6(35.29%)
Reference ranges (ng/mL)	Lower 12-137	75(25.42%)	5(15.15%)	4(33.33%)	5(29.41%)
	Higher 12-137	30(10.17%)	1(3.03%)	1(8.33%)	0
	Lower 6-239	38(12.88%)	0*	2(16.67%)	3(17.65%)
	Higher 6-239	11(3.72%)	0	1	0

Note: “M (IQR)” means the median (quartile); “(X ± SD” means the mean ± standard deviation.“*”, “P < 0.05” and “* *” means “P < 0.001”, a statistically significant difference.

Several key differences emerged in the group of patients who died. These patients were older, had a lower median dose of rivaroxaban, exhibited prolonged PT, and had higher CHA2DS2-VASc scores than those in the normal group. They also had a higher prevalence of hypertension and coronary artery disease (P < 0.05). However, no significant differences in rivaroxaban plasma concentrations or co-medications were observed between the death and control groups.

Using the reference ranges for laboratory monitoring of direct oral anticoagulants (DOACs) recommended by the International Committee of Hematology in 2021 for patients with atrial fibrillation and DVT, we explored the relationship between these ranges and clinical events (Douxfils et al., 2021). In the bleeding group, none of the patients had plasma levels <6 ng/mL, and the proportion of low-concentration patients was significantly lower than that in the normal group (P < 0.05). No significant differences were found in the plasma concentrations above the reference range. Owing to the relatively small sample size of patients with adverse events, these findings warrant further validation.

### 3.4 Correlation between rivaroxaban plasma concentrations and PT, TT, APTT, and D-dimer

**FIGURE 6:**
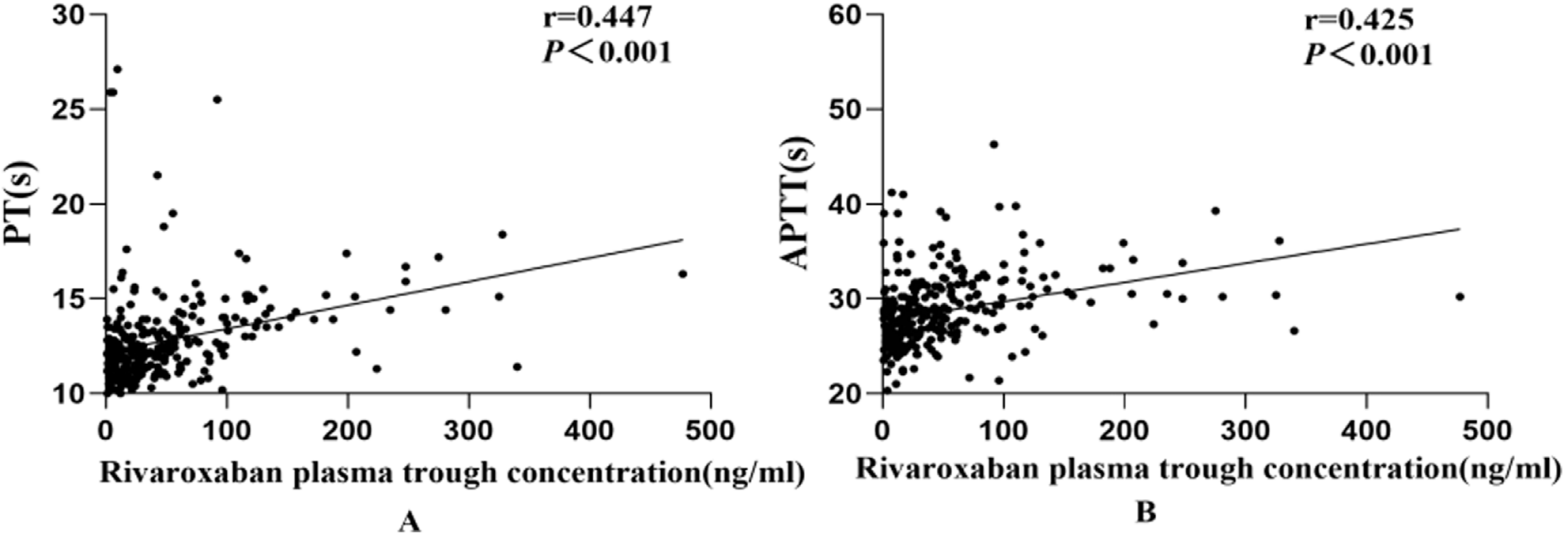
Correlation between rivaroxaban plasma through concentrations and PT **(A)** and APTT **(B)**.

Spearman’s correlation analysis revealed a moderately strong association between rivaroxaban trough concentration and PT and APTT (Figure 6). In contrast, no significant correlation was observed between the TT and D-dimer levels. The coefficient of determination (*R* ^2^) between rivaroxaban and PT was 0.447 (P < 0.001), whereas the *R* ^2^ value between rivaroxaban and APTT was 0.425 (P < 0.001).

## 4 Discussion

This study investigated factors associated with rivaroxaban exposure and clinical outcomes in real-world hospitalized patients. Age and renal function were significant predictors of rivaroxaban use. Patients who experienced bleeding events had significantly higher rivaroxaban exposure and a higher prevalence of malignancy. Patients with a history of bleeding or stroke are at an elevated risk of recurrence of bleeding or thrombotic events. Patients who died were typically older and had prolonged PT, higher CHA2DS2-VASc scores, and multiple comorbidities.

Since DOACs were introduced in the European Union in 2008, their global uptake for stroke prevention in patients with NVAF and for the treatment of DVT and pulmonary embolism has grown substantially. In particular, the use of rivaroxaban and apixaban has increased, and nearly half of patients taking DOACs are over 75 years of age (Stevens et al., 2021; Ibáñez et al., 2019). However, there is an ongoing debate regarding safety and need for dose adjustments in older patients. Recent studies have shown that exposure to rivaroxaban in older patients can vary widely, partly due to age- and renal function-related changes in pharmacokinetics (Edwina et al., 2023; Miklič et al., 2019; Gulilat et al., 2017). Consistent with earlier findings, we observed that patients with advanced age and renal impairment had significantly higher rivaroxaban plasma levels (Kubitza et al., 2010; Mueck et al., 2011). However, some studies have found no significant effect of age or sex alone on rivaroxaban pharmacokinetics (Kubitza et al., 2013). Large randomized controlled trials and population-based cohort studies have demonstrated that DOACs are safer and more effective than warfarin in older patients, even those over 75 years of age (Halperin et al., 2014; Chao et al., 2018).

Secondary analyses indicated that patients with bleeding had higher plasma levels of rivaroxaban and higher CDRs. Previous preclinical studies have demonstrated a correlation between serum rivaroxaban concentration and dosage, indicating a dose-dependent effect (Kvasnicka et al., 2017). Several previous studies have similarly reported that elevated DOAC trough concentrations may increase the risk of bleeding, whereas thrombotic events often occur in patients with lower trough concentrations (Lin et al., 2023; Testa et al., 2018; Lin et al., 2023). However, precise thresholds for bleeding and thrombosis remain undefined. To enhance patient safety, some authors have suggested monitoring plasma levels in patients at high risk of bleeding or thromboembolism. We also found that advanced age was associated with thrombosis and mortality. Because older individuals tend to have increased vascular fragility and declining organ function, they face increased risks of both bleeding and thrombosis (Lloyd-Jones et al., 2009; Andreotti et al., 2023). Evidence from large multicenter pharmacovigilance studies confirms that patients aged ≥75 years have an elevated likelihood of recurrent thrombotic events, bleeding, and subsequent mortality (Lauber et al., 2018; Kampouraki et al., 2021).

Our results further suggest that previous bleeding and stroke are associated with higher risks of recurrent bleeding and thrombotic events, respectively. Consistent with other studies, the history of bleeding has been linked to recurrent major bleeding (Lin et al., 2023). However, few studies have investigated stroke history as a risk factor for stroke recurrence or systemic embolism in hospitalized patients. Other investigations in older populations have identified unprovoked VTE and proximal DVT as risk factors for recurrent VTE (Lauber et al., 2018). The present results add to the evidence that a history of stroke predisposes patients to further stroke or systemic embolism, highlighting the need for cautious management in these individuals.

In real-world settings, management of patients with bleeding varies widely. In this study, we provided observational data on hemorrhage management. An observational study on the therapeutic management of patients with severe hemorrhage revealed that prothrombin complex concentrates were widely used but varied according to the site of bleeding and DOAC concentration (Albaladejo et al., 2017). The study also demonstrated that patient mortality was associated with the severity of the bleeding site (Albaladejo et al., 2017). Given the complexity of managing severe hemorrhage, larger studies are needed to identify optimal diagnostic and therapeutic approaches and further elucidate prognostic outcomes.

Several bleeding events in this cohort were associated with malignancy, consistent with prior evidence indicating that rivaroxaban use in cancer patients may increase the risk of gastrointestinal bleeding (Gu et al., 2020; Wang et al., 2018). Accordingly, current guidelines recommend apixaban or low-molecular-weight heparin as the preferred choice for anticoagulation in cancer-associated thrombosis (Streiff et al., 2018). Although the present and previous studies did not detect a statistically significant impact of CYP3A4 or P-gp inhibitors on rivaroxaban exposure at the group level (Lin et al., 2023; Kampouraki et al., 2021; Zhang et al., 2022), isolated case studies (e.g., the interaction with levetiracetam (Paciullo et al., 2020)) highlight the importance of individualized evaluation of DDIs.

We also found that the mortality group had a higher prevalence of hypertension and coronary artery disease. Both conditions are highly prevalent and cause significant morbidity and mortality, particularly among older adults in Asia (Lewington et al., 2002; North & Sinclair, 2012; Lacey et al., 2018; Lawes et al., 2003). These findings demonstrate the need to carefully select anticoagulation regimens for older patients with multiple comorbidities to minimize adverse outcomes. Although we did not observe any significant association between heart failure and clinical outcomes, future studies with larger sample sizes are warranted, given previous research indicating that heart failure may influence DOAC pharmacokinetics via reduced renal function (Lin et al., 2023).

This study found no associations between sex, BMI, co-medications, and clinical outcomes. While some studies align with our results (Halperin et al., 2014; Russo et al., 2021; Guarascio et al., 2023), others have reported contrasting findings. For example, a meta-analysis suggested a lower risk of venous thrombosis in women than in men (Douketis et al., 2011). Piran et al. found that 21% of individuals with a body weight >120 kg had DOAC peak concentrations below the reference range (Piran et al., 2018). Another study by Krauss reported a BMI-sex interaction, revealing that obese or morbidly obese men exhibited a higher incidence of significant bleeding, whereas women showed the opposite trend (Krauss et al., 2019). These discrepancies warrant further investigation.

Our study had several notable advantages. First, this study was based on real-world data, comprehensively collecting detailed information on a large number of hospitalized patients, particularly data on the older population. This study provides a valuable resource for evaluating the practical application of rivaroxaban in clinical practice. Second, by measuring the plasma trough concentration of rivaroxaban, we directly correlated drug exposure levels with clinical characteristics and outcomes, thereby offering a scientific basis for individualized dosing. These advantages not only endow our study with high clinical utility but also provide important references for optimizing anticoagulation therapy strategies in the future; however, certain limitations should be noted. First, because this study focused on rivaroxaban (the most commonly used DOAC at our facility), we did not compare rivaroxaban with other DOACs (e.g., apixaban), which recent studies suggest may offer a favorable safety profile. Future investigations should compare the efficacy and safety of these anticoagulants in similar patient cohorts. Second, the small sample sizes and brief follow-up periods constrained subgroup analyses for thrombotic and fatal cases. Larger-scale studies with extended follow-up are needed to clarify these relationships in older patients who require anticoagulation therapy, and the heterogeneity of dosing regimens may introduce confounding effects. To address these limitations, subsequent analyses focused on the once-daily dosing group, utilizing dosage, rivaroxaban plasma trough concentration, and CDR as key parameters to further evaluate pharmacokinetic variability and clinical implications.

## 5 Conclusion

Age and renal function significantly affected rivaroxaban exposure in hospitalized patients. Higher rivaroxaban exposure, history of bleeding, and malignancy were significantly associated with bleeding events, whereas advanced age and history of stroke were associated with thrombotic events. Patients who died tended to be older, exhibit higher CHA2DS2-VASc scores, have impaired coagulation, and present with multiple chronic conditions. We recommend routine monitoring of plasma rivaroxaban concentrations in older adults, particularly in those with multiple comorbidities or reduced hepatic, renal, or coagulation function, to help guide clinical decision-making. Future studies should seek to define the threshold values for bleeding and thrombosis, especially in older adults.

## Data Availability

The raw data supporting the conclusions of this article will be made available by the authors, without undue reservation.
